# Conversation-Based Information Delivery Method for Facility Management

**DOI:** 10.3390/s21144771

**Published:** 2021-07-13

**Authors:** Kuan-Lin Chen, Meng-Han Tsai

**Affiliations:** Department of Civil and Environmental Engineering, National Taiwan University of Science and Technology, Taipei 106335, Taiwan; m10705504@mail.ntust.edu.tw

**Keywords:** building information modeling, facility management, chatbot, natural language processing, ontology

## Abstract

Facility management platforms are widely used in the facility maintenance phase of the building life cycle. However, a large amount of complex building information affects facility managers’ efficiency and user experience in retrieving specific information on the facility management platform. Therefore, this research aims to develop a conversation-based method to improve the efficiency and user experience of facility management information delivery. The proposed method contains four major modules: decision mechanism, equipment dataset, intent analysis, and knowledge base. A chatbot prototype was developed based on the proposed method. The prototype was then validated through a feasibility test and field test at the Shulin Arts Comprehensive Administration Building in Taiwan. The results showed that the proposed method changes the traditional information delivery between users and the facility management platform. By integrating natural language processing (NLP), building information modelling (BIM), and ontological techniques, the proposed method can increase the efficiency of FM information retrieval.

## 1. Introduction

Sustainable operation is a key issue in a building’s life cycle [1]. Studies have been conducted to develop new algorithms, technologies, or strategies to improve the building operation and management process, such as facility management [2,3,4], defective building maintenance [5,6], and functional service life prediction [7,8,9,10]. Within these years, information technology (IT) and building information modelling (BIM) technologies have been gradually applied to the field of facility management (FM). Platforms, webpages, computer software, or mobile applications have been developed to help facility mangers or contractors collect, process, and reveal the facility-related data [3]. By utilizing BIM technologies, these tools can help the manger to manage facilities in a visualized and structuralized way [2,4]. Contractors can also use these tools for their daily operation or maintenance.

Although tools or systems have already been developed for facility management, the information delivery method is rarely discussed in previous studies. In the FM phase, a single building may contain a huge amount of facility-related data, especially for buildings like recreation centers, schools, and dormitories. The lack of an effective information delivery method makes it hard to retrieve information from existing BIM models and thus reduces the efficiency of the overall maintenance process. Contractors may have extra investments in training their engineers to get familiar with different platforms’ UI and operation processes whenever they get new contracts from different building owners. Therefore, this research will focus on how to deliver the facility information to related personnel in a more effective and intuitive way.

With the increasing usage of communication platforms and the growth of artificial intelligence technologies, using a chatbot as an information delivery system has garnered lots of attention in different fields. Tsai et al. [11] pointed out that conversation-based agents can interact with users in natural language and extract their real intentions during the interaction process. Users can easily retrieve the required information through natural languages. Wu et al. [12] mentioned many examples of chatbots used in different fields. These chatbots assist users in retrieving information by using natural language conversations.

This study aims to develop a new information delivery method to solve the low retrieval efficiency and the complex interface of traditional facility management platforms. The developed method focuses on (1) intent analysis for the natural language process and (2) ontology for structuralized BIM FM information. Combining these two technologies’ advantages, the developed method will serve as a communication bridge between the facility management platform and users. The method would be implemented as a chatbot system and evaluated using real example FM tasks at the Shulin Arts Comprehensive Administration Building of the New Taipei City Government.

## 2. Literature Review

BIM technology has been gradually applied in different stages of the building life cycle, resulting in a massive amount of model information compared with the past. Therefore, how to efficiently manage and apply BIM model information has gradually attracted attention [13]. In recent years, many studies have focused on the retrieval of BIM data. These studies include the FM platform acting as a bridge to provide users with retrieval information, the use of the ontological approach to classify BIM information, and different methods proposed for retrieving model information. This section is divided into four areas of previous research: BIM in facility management, challenges in retrieving BIM information, applications of ontology in BIM, and conversation-based information delivery systems.

### 2.1. BIM in Facility Management

Throughout a building’s life cycle, maintenance and operating costs during its useful life could amount to many times its construction cost. Facility engineers rely on integrated information to support their daily tasks and decisions. Akcamete et al. [14] suggested that the lack of reliable facility information to predict breakdowns and proactively maintain the facility will result in passive maintenance. Therefore, effective maintenance and management of buildings can significantly reduce operating costs. In previous studies, many researchers have discussed the advantages and benefits of BIM application in the FM stage of buildings. For example, BIM sends initial data to the FM system and provides other visualization and analysis functions during the FM phase [15] which improve the existing manual handover process and the accuracy of FM data, and speed up the retrieval of data [16,17]. BIM in FM provides a reliable facility information database, 3D spatial information, and integrated views [18]. Some studies show that the application of BIM improves the efficiency of the delivery process and the accuracy of information recording. These advantages can help users achieve more effective reactive maintenance and preventive planning [19,20]. Edirisinghe et al. [21] evaluated the existing research and summarized the benefits of BIM, especially a BIM-enabled FM implementation process based on innovation diffusion theory. Yalcinkaya and Singh [22] scrutinized the nature of BIM and FM. Their analysis focused on the value-adding potential of BIM, with the results indicating the benefits of BIM for FM. Based on these previous studies, BIM plays an important role in FM and has benefits in terms of data integration, visualization, and delivery. As a result, BIM-based FM technology has brought many advantages and improved the efficiency of data management.

### 2.2. Challenges in Retrieving BIM Information

Although BIM can play an important role in FM, some challenges have also emerged. BIM stores model data at various stages in the building life cycle, resulting in a large amount of complex information. Nepal et al. [23] suggested that there are still tremendous challenges in receiving construction-specific information from BIM. This limits some applications of BIM models in construction and other downstream processes. The continuous integration of BIM would make it difficult for users to obtain the required data on a mobile device with limited space for interaction [24]. There is an important outstanding question regarding how to filter redundant information and extract specific details from complete BIM models [25,26]. BIM can clearly represent component attributes and relationships between components. To help users access information effectively and instantaneously could minimize the time required to retrieve information and help prevent equipment maintenance personnel from making poor decisions in the absence of information [27]. Informatization in the construction industry is lagging and has several major issues. These issues include difficulties in the sharing and integration of information caused by varying data formats of building information [28], BIM as a product-centric information database that lacks domain semantics [29], and lack of interoperability between BIM tools [30]. While the open BIM Industry Foundation Classes (IFC) schema can define object relationships, it lacks connections between building elements and more detailed attributes. IFC data is intended for the creation and exchange of profile data but is not tailored for various queries, so it is difficult to retrieve relationships and data attributes that are clearly defined or implied in the model [31]. The rapid expansion of accumulated and stored building data means that semantic data analysis in the FM field is essential. From past research, many reasons for inefficient retrieval can be found, with the primary reason being the lack of a relationship and data sharing between components, which prevents users from effectively obtaining the desired data.

### 2.3. Applications of Ontology in BIM

In order to solve the inefficient data retrieval problem, ontological methods have been proposed. Ontology can be used to formally express knowledge that includes concepts, relationships, and rules in a specific field. For example, ontology can hide the complexity of the product model from the end-user [32], and the use of graphical data structures for information modeling helps to integrate information from different sources [33]. Lee et al. [34] pointed out that using ontology could accurately identify working conditions and automatically infer appropriate work items. There have been some previous studies on how to apply ontology methods to BIM. For example, a framework based on IFC and COBie data could solve the problem of interoperability of information between BIM and FM. This framework enables the facility maintenance information to be successfully mapped from the BIM model to the COBie data and be imported into the FM system through an ontological approach [35]. Some new approaches based on ontology facilitate the management of context-sensitive construction information stored in different documents [36], achieve semantic interoperability [37], and are built with Web Ontology Language (OWL) to help users directly query the IFC model in the XML format through an information retrieval system [38]. A semantic retrieval system framework based on a construction project risk domain ontology can improve the semantic expression of information [39]. From the many research publications described above, the application of ontology to BIM is becoming increasingly widespread. Ontological technologies form connections between component data, especially in the field of FM, and thus the relationship between equipment and space can be clearly defined. Ontological technologies will bring new advances to data retrieval methods.

### 2.4. Conversation-Based Information Delivery System in Variuos Fields

In recent years, due to the rapid development of technologies such as natural language processing, machine learning, and speech recognition, the interaction between users and machines is becoming increasingly diverse. Nuruzzaman and Hussain [40] suggested an advantage in conversational robots in attracting users to dialogue and these investigators believed that chatbots would become the most preferred form of communication for businesses in the future. In addition, some studies have used a chatbot as a conversation-based system to achieve various purposes. Table 1 summarizes different chatbot systems that have been developed. Oh et al. [41] built a dialogue system for mental healthcare services. High-level natural language understanding and emotion recognition approaches were used for psychiatric counseling. To address the drawbacks of traditional chatbots, such as the need of learning specific languages, OntBot was developed to ease the user interactions of using natural languages. By transferring ontologies and knowledge into a relational database, OntBot could provide seamless support of different application domains [42]. Vegesna et al. developed an ontology-based chatbot that can process e-commerce website queries from users to help users understand product details [43]. There are also some application cases of chatbots in the field of civil engineering. Ask Diana is a chatbot that was developed for disaster-related information management and delivery [10]. Tsai et al. used a fuzzy search algorithm to analyze users’ intents when they queried information through Ask Diana [44]. For instance, Tsai et al. [45] developed a disaster management decision support system developed for the Bureau of Mine in the form of a chatbot. Cho and Lee [46] proposed a daily construction report data management system by using chatbot. The chatbot is responsible for collecting and processing the required information through conversations with users and generates daily reports automatically. Chan and Tsai developed a question-answering dialogue system for emergency operations through named entity recognition (NER) and question classification (QC) [47]. McArthur et al. used supervised machine learning models to perform the classification for work orders generated from occupant complaints [48].

Although chatbots have been widely used in different fields, implementing it into facility management applications has rarely been discussed. From the research results of previous studies, the author found that the chatbot has three major advantages that makes it suitable and promising as a facility information delivery solution. Frist, the chatbot has the ability to handle large amounts of complex data [44]. In the operation phase, a single building may contain various and tedious information. It is essential for an information delivery method to process all the data and provide correct information to specific personnel. Second, the chatbot has high mobility compared to traditional platform-based systems [11]. A traditional platform-based system usually requires users to operate on personal computers or laptops. It makes it hard for contractors or facility managers to bring required information to facilities when conducting regular maintenance tasks. Being built on communication platforms which usually support mobile devices (e.g., smart phone, pads) will allow the related personnel to carry required information everywhere. Last, the chatbot can reduce the learning threshold of FM system by allowing users to query information by using intuitive user interfaces or natural languages [43,47].

## 3. Methodology

**Figure 1:**
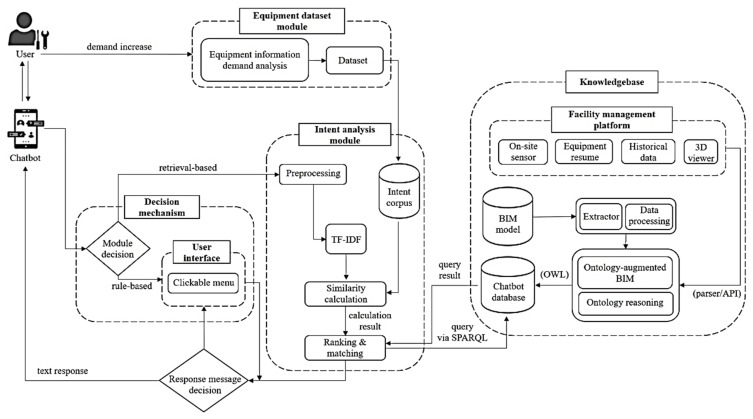
Conversation-based system architecture.

This research aims to develop a new information delivery method that provides the FM-related personnel with (1) an efficient approach to retrieve facility information through dialogue quires and (2) real-time observations of three-dimensional models. An overview of the proposed conversation-based system is shown in Figure 1. The system architecture of the proposed method contains four major modules: the decision mechanism, equipment dataset, intent analysis, and knowledge base. The decision mechanism module, equipment dataset module, and intent analysis module are mainly designed to enable the conversation-based system to understand the user’s true query intent. The knowledge base is important for processing BIM data and serves as the main source of response sentences for the information delivery.

First, the equipment dataset module is designed to define the search scope of the conversation-based method clearly. The maintenance of facilities in the same building may be performed by several users with different professional backgrounds, such as fire protection engineers, mechanical and electrical engineers, and building managers. Users with different professional backgrounds have different requirements for BIM data. Therefore, building an equipment dataset will be an important process to determine the search scope of the conversation-based information delivery without clear needs from users. This module will be used to summarize the list of items that need to be retrieved. Then these lists will form a dataset, and the dataset will be imported into the intent corpus in the intent analysis module. The decision mechanism determines whether the user’s interaction with the system is rule-based or retrieval-based.

When retrieval-based behavior is triggered in the decision mechanism module, the intent analysis module would process the user’s natural sentences and execute the intention. The results will be used as a reference for the method to reply to users’ queries. The knowledge base uses ontological techniques to process the large quantity of BIM information from the FM platform and BIM model. The processed data will be stored and used as the data source for similar calculations in the intent analysis module. The following subsections describe details for each module, respectively.

### 3.1. Knowledge Base Module

The knowledgebase module will play the role of data integrator and information provider. After analyzing the user intent, a request will be sent to the knowledge base module to get the information that matches the user’s intent. During facility managing processes, the information delivery method may require dealing with a huge amount of information, such as the BIM model, on-site sensor, equipment resumes, historical data, etc. To structuralize all the data, an ontological method is proposed in this research. The ontological method combines the ontology and markup language, which allows an undefined object to be structuralized. This research applies the ontology method to the semantic network for the data search. The hierarchical relationship of the ontology would define the object. Then, these data would be used to perform data reasoning and thus creates links to each data.

**Figure 2:**
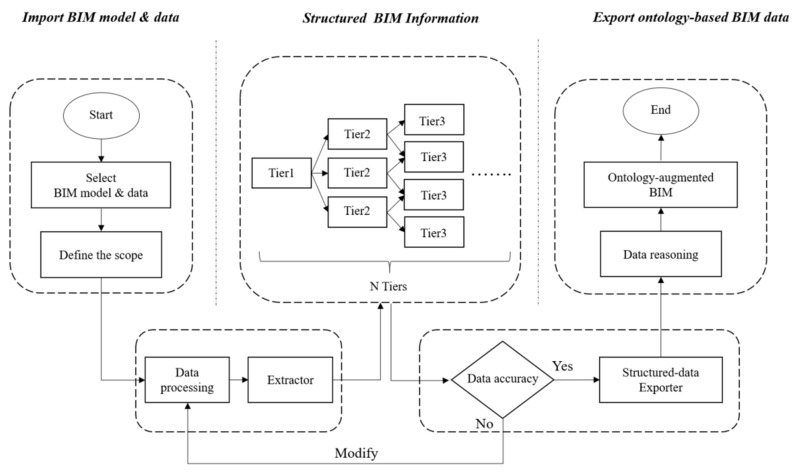
The process of structuring BIM data.

As shown in Figure 2, the developed ontological method contains three steps: importing the BIM model and data, structuring BIM information, and exporting ontology-based BIM data.

1. Importing the BIM model and data: In this step, the required information will be extracted from BIM data according to the users’ needs. The results of the personal interviews conducted in the equipment dataset can also be the reference to determine what kinds of data should be included. Then, the extracted will be pre-processed, including unnecessary information removal and redundant words or symbols elimination.
2. Structuring BIM information: In this step, the proposed data will be structuralized into different tiers. The first tier will be the widest range, with the tiers gradually narrowing. Taking a building as an example, the first tier may be the building itself; the second tier may be the floor in the building; the third tier may be the room number of each floor, and so on. To structuralize the BIM data, an extractor, which can filter the data by following the sequence of the category, the floor, and the room of the model, was developed. Such an extractor can help efficiently organize the data of the entire building into different tiers.
3. Exporting ontology-based BIM data: In this step, the structured data will be reasoned using the ontological tools to create links between the data. Taking an object placed in space as an example, if its position is described from an object’s viewpoint, a link will be “this object is in space”. Conversely, when describing the position of an object in terms of space, the link will be “there is an object in this space”. The ontological tool can link the component data with spatial relationships and thus construct the ontology-based data.

### 3.2. Equipment Dataset Module

The equipment dataset module provides a dataset based on the needs of users to define the searching scope of the conversation-based information delivery method. In the FM phase, the users may have different professional backgrounds, such as fire protection engineers, mechanical and electrical engineers, and building managers. Those users will have different information requirements and different ways to query the FM information according to their professions. Therefore, it is important to build a dataset based on users’ needs. Such a dataset can both define the searching scope and be the intent corpus for user intent analysis.

**Figure 3:**
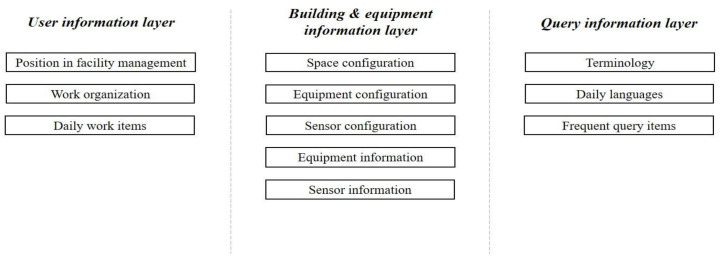
Structure of the equipment dataset.

To deal with the queries from users with different professions, this research designed the dataset with three layers: user information, building and equipment information, and query information (Figure 3). The user information layer is designed to collect information about the users, including their position in facility management works, work organization, and daily work items. The building and equipment information layer collects building and equipment component information, including space configuration, equipment configuration, sensor configuration, equipment information, and sensor information. The query information layer contains the terminology, daily languages, and frequent query items that users might use.

To process the natural languages entered by different users, the dataset should be collected based on the defined three layers. For the building and equipment information layer, the data can be collected from engineering drawings, existing BIM models, or facility management platforms. Since the data in the user information and query information layer may vary with different users, it is essential to collect the data based on users’ needs who may use the conversation-based system for their daily works. The data can be collected through either personal interviews or questionnaire surveys.

**Figure 4:**
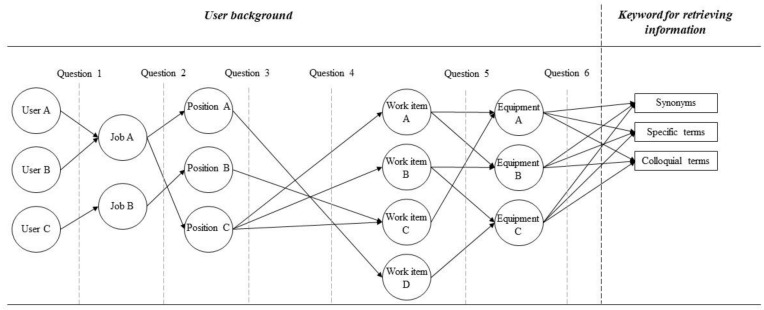
Contents of the user interview questions.

For the user information and query information layer, this research also designed a questionnaire to collect real users’ data. The content of the questionnaire is divided into two parts with 6 questions (Figure 4). The first part is to collect the user’s background, including job position, professional roles, daily work items, facilities operated, and facility information frequently retrieved. Besides background information, it is also important to collect the keywords used to query the information. Thus, the second part of the questionnaire is to collect the synonyms, specific terms, or colloquial terms frequently used by the user to query the information. Based on these two parts, this research developed the following six questions for the questionnaire:

Question 1: What is the user’s occupation and years of experience?
Question 2: What is the user’s position in the organization?
Question 3: What is the user’s work item in their job?
Question 4: What are the user’s daily work tasks?
Question 5: What kind of facility information do users frequently retrieve?
Question 6: What are the frequently used terms for users in their daily work?

### 3.3. Intent Analysis Module

After setting up the decision mechanism and building the equipment dataset, the third module, intent analysis, was developed to process the user’s natural languages. In the developed intent analysis module, an intent corpus is constructed to store various sentences that represent different user intents. The intents in the corpus are obtained from the equipment dataset module. These sentences may change dynamically based on who the user is and how often the task is quired. Besides the intent corpus, the intent analysis module uses three major steps to process the input from the user.

The first step is data preprocessing. In this step, the sentences entered by the user are processed to extract the keywords and eliminate unnecessary words or special symbols. To eliminate the redundant or unnecessary words, a word and symbol corpus is first built to store those words or symbols that need to be deleted. When a sentence is entered, every word and symbol in the sentence is checked to see whether the sentence contains any words or symbols defined in the word and symbol corpus. If the sentence contains such words or symbols, those words or symbols will be deleted. For example, when the user enters “I want to search the brand of the pump on the second floor”, the word “I”, “want”, “to”, “search”, “the”, “of”, “the”, and “on” will be classified as redundant words and deleted. Only “brand”, “pump”, “second”, “floor” will be used for the subsequent analysis.

The second step is similarity calculation. This step is developed to check the similarity between the keywords from the user’s input and the pre-defined intent corpus. The term frequency-inverse document frequency (TF-IDF) [49] method is utilized to convert each word in a sentence into a vector. TF-IDF is a statistical method used to evaluate the importance of a word in a sentence. The method consists of two parts: word frequency calculation (TF) and inverse document frequency (IDF). The TF-IDF formulation is given by

(1) $$tf_{w,D_{i}}=\frac{count\left(w\right)}{\left|D_{i}\right|}$$

(2) $$idf_{w}=log\frac{N}{1+\sum_{i=1}^{N}I\left(w,D_{i}\right)}$$

(3) $$tf-idf_{w,D_{i}}=tf_{w,D_{i}}\times idf_{w}$$

- $tf_{w,D_{i}}$: the frequency at which a particular entity name (w) appears in a sentence ($D_{i}$).
- $count\left(w\right):$ the number of occurrences of the entity name (w).
- $D_{i}$: the number of all words in a sentence.
- N: total number of statements in the corpus.
- $I\left(w,D_{i}\right)$: whether the statement $D_{i}$ contains the entity named in the corpus (if $D_{i}$ contains the entity name, then the value equals 1. Otherwise, the value will be 0).

The TF-IDF method would be applied to both the sentence entered by the user and sentences in the intent corpus to find the important keywords. Next, the cosine similarity method [50] is used to determine the similarity of the user inputs and the keywords in the corpus. The cosine value between the statement input by the user and the statement in the intent corpus can be calculated by:

(4) $$cos\;\theta =\frac{\sum_{i=1}^{n}\left(A_{i}\times Bi\right)}{\sqrt{\sum_{i=1}^{n}{\left(A_{i}\right)}^{2}}\times \sqrt{\sum_{i=1}^{n}{\left(B_{i}\right)}^{2}}}$$

where $A_{i}$ and $B_{i}$ are the frequency of each word in the user statement and the frequency of each word in the intent corpus, respectively, after being processed by the TF-IDF method. The higher cosine value represents that $A_{i}$ and $B_{i}$ have higher similarity. Based on this method, the similarity (cosine value) between the user input and each sentence in the intent corpus can then be calculated and stored as a series of similarity scores.

**Figure 5:**
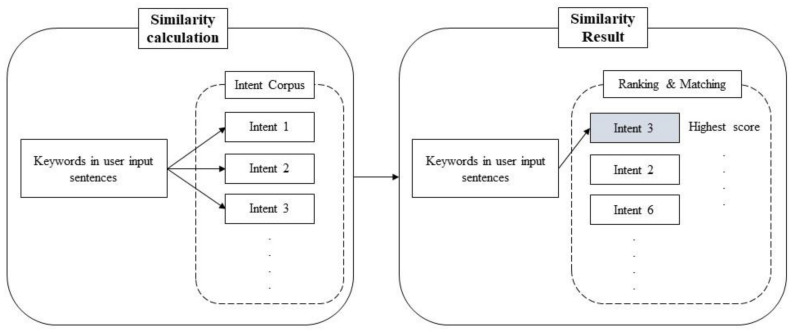
Schematic diagram of the ranking and matching process.

The last step is to rank and match the user input with the intent corpus based on similarity calculations. First, the similarity scores calculated in the similarity calculation step will be sorted from high to low (Figure 5). A constant number is set to be the threshold to avoid mismatching of the user intent. If the highest similarity score does not exceed the threshold, the user should be asked to re-enter a more precise query. Conversely, the intent with the highest similarity score with the user input will be selected as the closest intent and sent to the knowledge base module for information retrieval.

### 3.4. Decision Mechanism Module

In the conversation-based system, two approaches, rule- and retrieval-based, are commonly used to query the information. The rule-based approach provides a user interface, such as buttons and menus, to query the information by following the designated rules. The retrieval-based approach allows the user to query the information by entering the natural language. The proposed method uses both the rule- and retrieval-based approach for the user to query the FM information. The decision mechanism is developed to detect which approach should be triggered and how to reply to the user accordingly.

**Figure 6:**
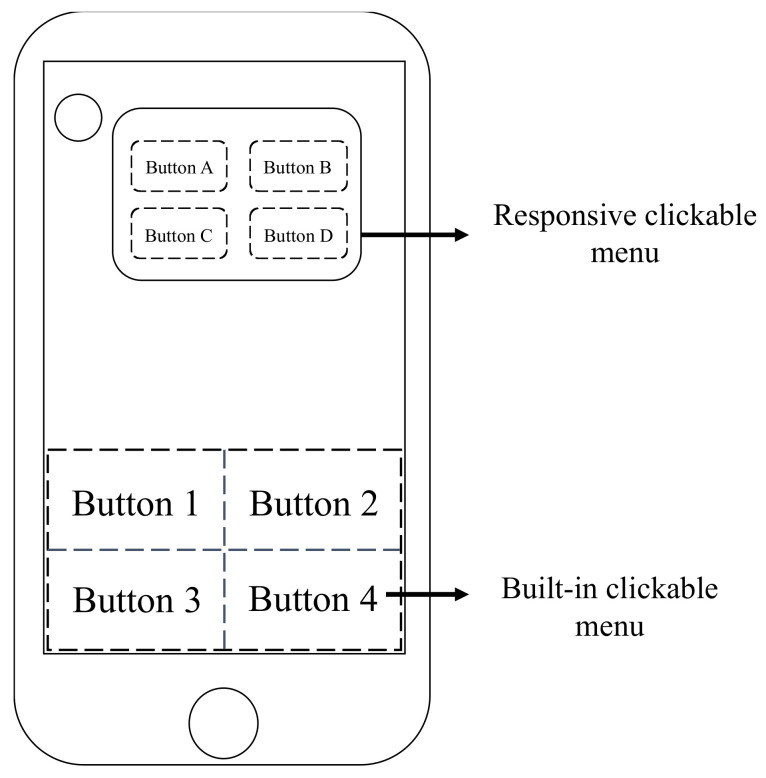
The developed method uses a clickable menu as the interface of the rule-based querying approach.

In the rule-based approach, this research provides the user with an intuitive operation interface and manually writes the statements that the user often enters as a rule for design. As shown in Figure 6, a built-in clickable menu is included in the user interface. The clickable form on the main page is designed with features that users often use. In this way, the user can quickly retrieve information by clicking a button. Messages from the dialogue system can also have clickable menus, and the type of clickable menu will change according to the user’s search intent. Each button in the user interface carries a rule-based statement. When the user clicks the button, the system will determine that a ruled-based approach is triggered and provide corresponding replies.

**Figure 7:**
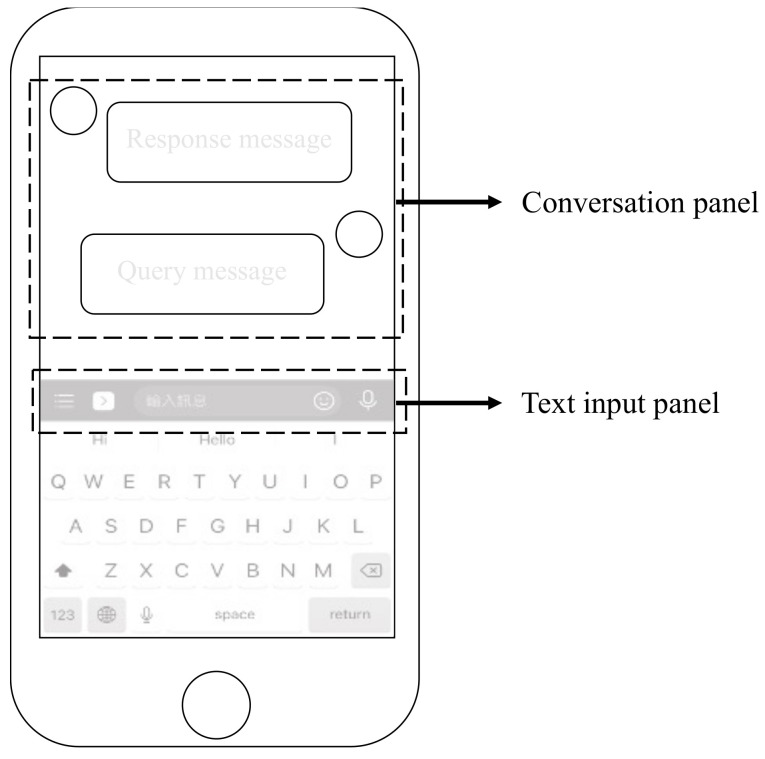
The developed method allows the user to input natural language through the text input panel.

The retrieval-based approach is triggered when the user queries the information by entering the natural language. As shown in Figure 7, the retrieval-based user interface includes a conversation panel and a text input panel. All response dialogues are recorded in the conversation panel, and users can enter text in the text input panel. The retrieval-based module will transmit the user’s statement to the intent analysis module for subsequent processing and analysis.

## 4. Real Case Implementation and Chatbot Prototyping

To implement the developed FM information delivery method, this research used a government administration building in Taiwan, the Shulin Arts Comprehensive Administration Building of the New Taipei City Government, as a real case example and developed a chatbot prototype. The implementation of the proposed method involves dataset definition, knowledge base construction, and chatbot prototyping.

### 4.1. Equipment Dataset Definition

To define the scope of the delivery method, user interviews were conducted using the designed questionnaire. Two professionals who have more than eight years of experience in facility management were interviewed. Their positions are general employees, and their jobs are to maintain and monitor all the equipment in the building. Each day, they have to inspect some specific equipment and sensor values. In addition to the content of the work, they also pointed out some challenges in daily operations. The results of the interview are as follows:

- The facility manager needs to monitor the value of each sensor every day and if an abnormal value is received, preliminary maintenance is required. If the problem cannot be solved, contact the facility contractor, such as the equipment provider or manufacturer.
- The information frequently retrieved by the two facility engineers includes the equipment full code, operating status, any peculiar smell, cleanliness of the environment, electricity usage, etc.
- Facility engineers need to go to each floor every day to inspect various equipment including the air-conditioning facility, ice water host, electric meters, etc.
- In the current operating procedures, the inspection records and error codes of various equipment are paper based, resulting in the accumulation of a large number of paper records.

The structure of the equipment dataset was then defined based on the results of the interviews. The structure of the dataset contains five columns: building name, floor list, room list, equipment list, and attribute list (Table 2). The first column stores the name of the target building. The second column stores all floors of the building. The third column stores the room list for each floor. The equipment list column stores the equipment in each room, including exhaust fans, cooling water towers, air conditioners, pumps, flow meters, ice water machines, and flow meters. The last column stores each piece of equipment’s attributes, including full code, location, inspection records, error codes, and detailed equipment information.

After defining the structure of the equipment dataset, the related FM information was collected based on the five columns of the dataset. According to this structure, this research collected the related data of the administration building from (1) the BIM model, (2) the existing FM platform used by the FM personnel, and (3) the engineering drawings.

### 4.2. Knowledge Base Construction

**Figure 8:**
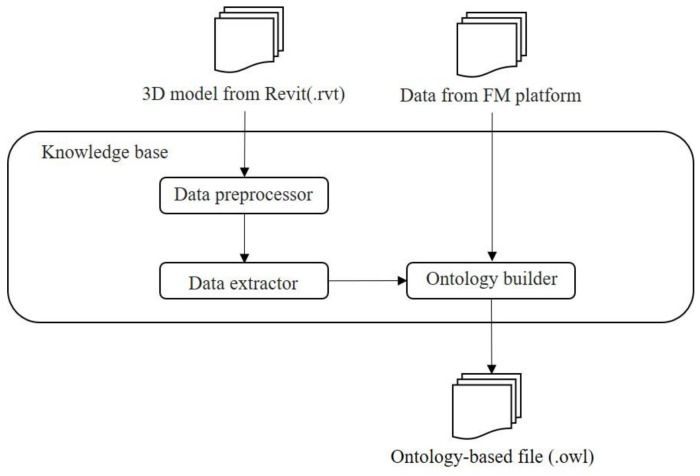
The workflow of knowledge base construction.

To structuralize the BIM data stored in the equipment dataset, a knowledge base for the administration building was constructed. Figure 8 shows the workflow of constructing the knowledge base. The knowledge base takes the data from two sources: the three-dimensional model from Revit (.rvt) [51] and the data extracted from the FM platform (.xlsx). To extract information from the BIM model, a data processor and extractor were developed. Two APIs were also developed to extract the information from the existing FM platform. An ontology builder, Protégé [52,53], was utilized to construct the knowledge base. Finally, the processed data was exported in ontology format files (.owl).

#### 4.2.1. BIM Data Preprocessor and Extractor

To solve the problem of null values, long string, and lack of attributes in the BIM model, this research developed a data preprocessor using C# [54] and Revit API [55]. The preprocessor assigns a “null” string when there is no value in the attribute field. When it detects that the string in the attribute field is too long, the preprocessor will process the string based on the composition rules. If there is a lack of specific attributes, the attributes will be added by the Revit API automatically. With the preprocessor, the correctness of the data transferred to the ontology builder can be ensured.

After preprocessing the data, a data extractor was developed to extract the model attributes in Revit. The extractor was developed using Python [56] and Dynamo [57], a visual programming tool. The extractor is responsible for extracting five categories of information in the BIM model, including the position information of the model, the size information of the model, and the customized information, such as the manufacturer, cost, voltage, and so on. Then, the extractor stores the data in an ontology framework in the ontology builder.

#### 4.2.2. APIs for FM Data Extraction

To connect the data of the existing FM platform, two APIs were prepared using Python scripts. The first API is used to extract facility information from the platform, including facility name, full code, floor information, location information, inspection records, error codes, detailed facility information, and so on. The other is used to receive on-site sensor data, including sensor name, senor value, and last updating time. The obtained information from either the FM platform or on-site sensor will be stored in the ontology framework for further uses.

#### 4.2.3. Ontology Builder

**Figure 9:**
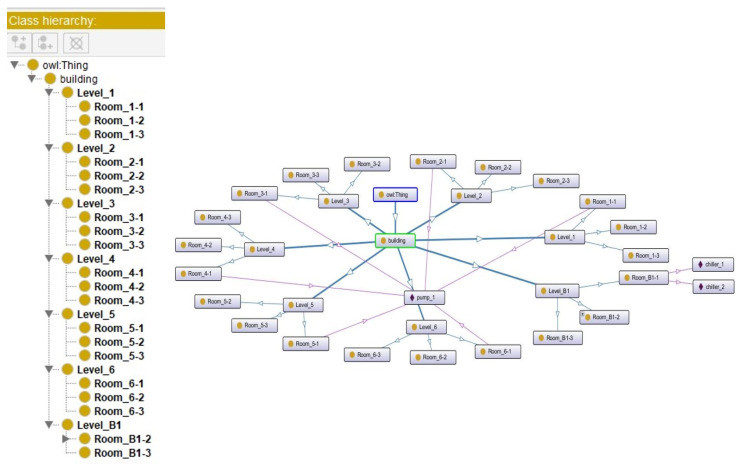
The ontology framework of the target building.

**Figure 10:**
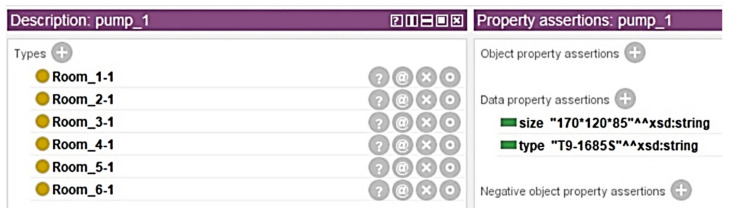
Example of the individuals in the ontology framework.

This research used Protégé, an ontology editing and knowledge management software, to construct the knowledge base. As shown in Figure 9, layers were built in the order of building, floor, and room. There are seven floors in the target building, including the ground floor. Different pieces of equipment are in each room, and each piece of equipment has its own attributes. For example, except for the ground floor, each floor contains three rooms. Every Room 1 of each floor has a pump. Room 1 on the ground floor has two chiller devices. Individuals with properties can be established in each layer in the ontological framework. As shown in Figure 10, the pump of each floor has the properties of size and type, and these properties can be flexibly added or deleted.

**Figure 11:**
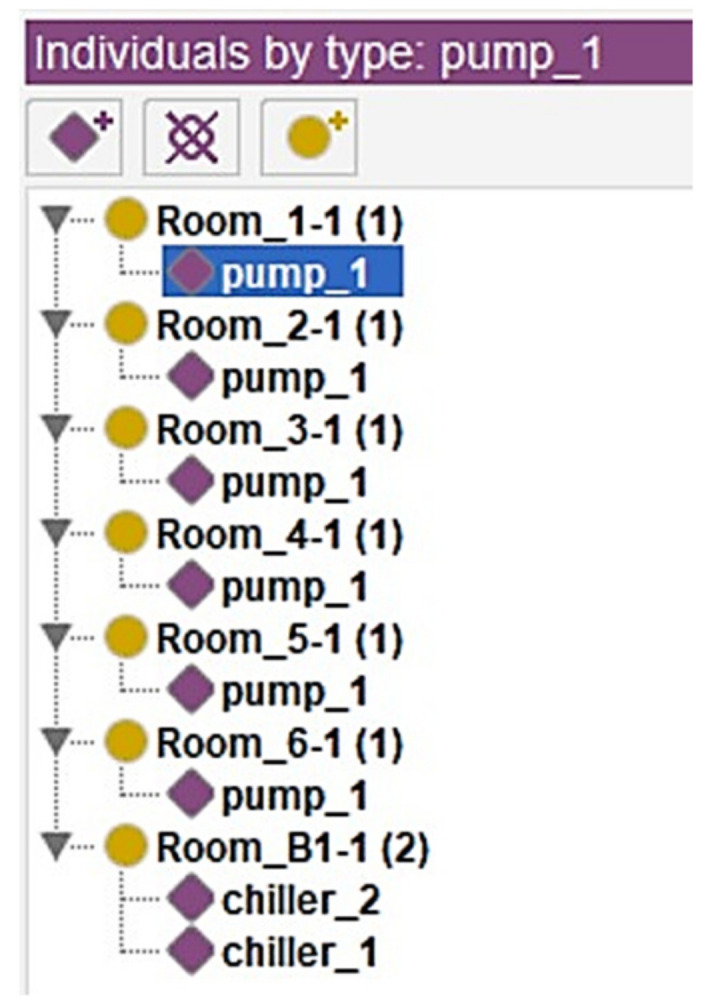
Example of the data reasoning results.

With the ontology framework, HermiT was used to conduct the data reasoning. HermiT is an ontology reasoner written using Web Ontology Language (OWL) [58]. By executing ontology reasoning, it will reclassify individuals and classes for the BIM data, which means creating the parent-child hierarchy of individuals and classes. After reasoning, each piece of equipment with its properties was assigned to the room and floor that it belongs to (Figure 11).

### 4.3. Chatbot Prototyping

**Figure 12:**
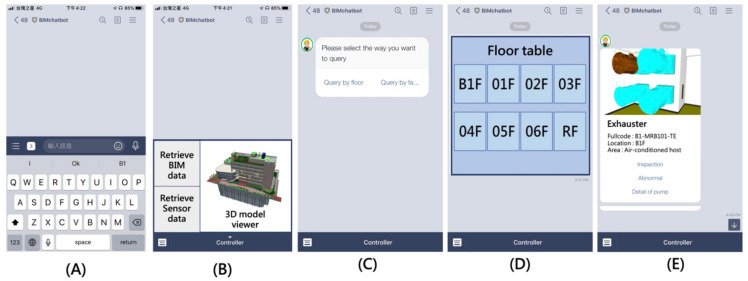
The main interface and menus of the chatbot: (**A**) the message input bar, (**B**) the rich menu, (**C**) confirm template, (**D**) imagemap message, and (**E**) carousel template.

A chatbot was prototyped based on LINE, an instant messaging platform with the highest market share in Taiwan [59], to deploy the developed information delivery method. The LINE chatbot provides two main interfaces for users to query the FM information through either rule- or retrieval-based methods. Figure 12A shows the “message input bar”, which allows the user to query the information by inputting natural language. A clickable rich menu, built by LINE’s API, was designed for the user to query the information through a rule-based method (Figure 12B).

Besides the main interfaces, three different interactable menus were designed for the user to retrieve the information more efficiently: confirm template, imagemap message, and carousel template. The confirm template menu provides binary options for the user to choose such as yes or no questions or query by floor or facility (Figure 12C). The imagemap message contains an image with multiple clickable areas. It was used to provide multiple options for the user. For instance, this research uses the imagemap message with a floor table of the building. The user can select the target facility’s floor location that they want to query by clicking the image (Figure 12D). The carousel template was used to reply to the user with multiple column objects with summarized information. Each column contains one picture, equipment name, and summarized information. Each column also has three buttons for the user to have further actions, including inspection of the equipment, report abnormality, or review detailed information. Figure 12E shows an example of how the exhauster information was revealed in the carousel template.

**Figure 13:**
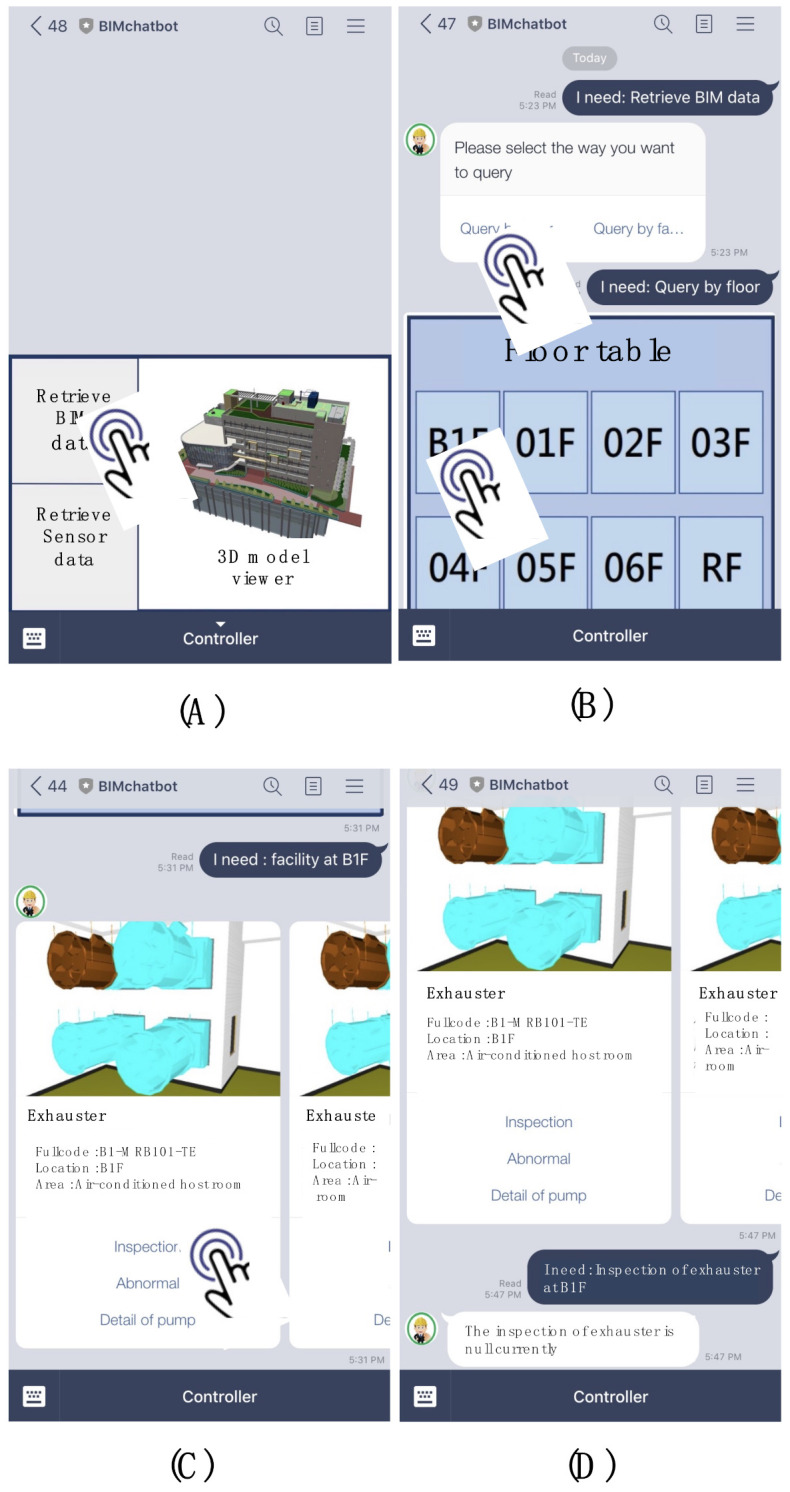
Example of the operation of the chatbot prototype using designed interface: (**A**) click Retrieve BIM data from the rich menu, (**B**) click Query by floor and select B1F from the imagemap, (**C**) find the designated facility and click Inspection, (**D**) the chatbot replies to the user with the inspection information.

For example, if the user wants to query the inspection information of an exhauster located on B1F, the user can first click the “Retrieve BIM data” in the rich menu of the main interface (Figure 13A). The chatbot will reply to the user using a confirm template to ask if they want to query the information by floor or facility. If the user clicks “Query by the floor” of the confirm template, the chatbot will send the message, “I need: Query by floor”, automatically and reply to the user with the floor table in the format of an imagemap (Figure 13B). After selecting B1F from the imagemap, the chatbot will list all the equipment locations on B1F by using the carousel menu. The user can find the exhauster on B1F by scrolling the carousel menu (Figure 13C). Last, the user should click the “Inspection” button in the “Exhauster” column to query the inspection information. The chatbot will send “I need: Inspection of exhauster at B1F” automatically and provide the information accordingly (Figure 13D).

## 5. Validation

To verify that the developed conversation-based delivery method can be utilized for facility management, a feasibility test and a field test were conducted. For the feasibility test, three scenarios were designed for three students with a civil engineering background to test whether the chatbot can successfully complete the task according to the repetitive work content of the facility manager. For field testing, we collaborated with the facility management team at the Shulin Arts Comprehensive Administration Building and tested a chatbot application for assisting their daily tasks.

### 5.1. Feasibility Test

For the feasibility test, we invited three master’s students with a civil engineering background who were familiar with using the Line platform. Each participant was required to test three scenarios using two different methods for the chatbot. The time spent to complete the task was recorded. If the task could not be completed, the reason was recorded. Finally, the participant’s experience with and suggestions for the chatbot were recorded. The following sections will describe the designed feasibility test and the results of the feasibility test.

#### 5.1.1. Design of the Feasibility Test

The content of the three scenarios and the two methods of an operating chatbot are described in detail in Table 3. The first scenario was to use the chatbot to retrieve facility information. Participants were required to use the chatbot to retrieve facility information, including a list of equipment on specific floors, inspection records, full code, and the description of specific equipment. The second scenario was to use the chatbot to retrieve on-site sensor information. Participants were required to use the chatbot to retrieve on-site sensor information, including the value and percentage of the thermal cumulative flow of the flow meter sensor on a specific floor. The last scenario was to use the chatbot to operate a 3D viewer. The participant was asked to use a chatbot to operate the 3D viewer and find specific facilities in the viewer. These three scenarios were required to be completed using two different methods for the chatbot. The first method was operating the chatbot by using the clickable menu. The participants were required to complete the task by only clicking the button or menu in the chatbot interface. The second method was to use natural language to operate the chatbot. Participants could only retrieve information by entering natural language and could not use any button or menu in the interface. The test results, operating experience and suggestions, and time spent by each subject were recorded.

#### 5.1.2. Results of the Feasibility Test

The results of the feasibility test were recorded and analyzed. Table 4 illustrates the feasibility test results. Three participants passed all three scenarios using both methods and the average time spent was approximately eighty seconds. Among the scenarios, the users spent the most time in Scenario 1. By observing the users’ operation of the chatbot, it was found that when users perform Scenario 1, they will take a long time to retrieve information as they are not familiar with the interface. Furthermore, the three participants all took a long time when using Method 2, as they may make mistakes when entering text or lack keywords in the input sentence. In addition, the experience and suggestions of the three users operating the chatbot to the perform tasks were recorded. These experiences and suggestions can be summarized in the following three comments:

1. The participants generally feel that using clickable buttons to retrieve information is more convenient and efficient than using natural language input. When attempting to use natural language to retrieve information, the participant may fail due to different language habits and keywords.
2. The model displayed in the 3D viewer can be simplified to display only important structures and equipment to solve the problem of operating complex models on a small phone screen.
3. The model in the 3D viewer should make more contributions. For example, the model can be combined with the value of the sensor to achieve the effect of numerical visualization, allowing users to more intuitively understand the operating status of the device.

### 5.2. Field Test

For the purpose of a real case study, we cooperated with the facility management team at the Shulin Arts Comprehensive Administration Building of the New Taipei City Government in Taiwan. The Shulin Arts Comprehensive Administration Building has ten floors, with three floors underground and seven floors above ground. Four participants were invited to participate in the field test: two facility engineers of the Shulin Arts Comprehensive Administration Building and two engineers who work for facility management platform companies. The following sections will detail the design and results of the field test.

#### 5.2.1. Design of the Field Test

For the design of the field test, thirteen pieces of equipment and six sensors in the Shulin Arts Comprehensive Administration Building were used as the test targets. This equipment includes two ice water machines and a pump located on the ground floor, flow meters, air conditioning units, and flowmeter sensors on the first to sixth floors, six ventilators on the sixth floor, and two cooling towers on the top floor. Table 5 shows the design of the field test, with three pieces of equipment and four sensors selected from the thirteen pieces of equipment and six sensors as the test questions. For the content of the field test, subjects were required to use three different methods to execute three scenarios. The first method was to use the existing online facility management platform, the second method was to use the clickable menu in the chatbot to execute the task, and the third method was to use natural language with the chatbot to execute the task. The three scenarios were the retrieval of facility information, retrieval of on-site sensor information, and operation of the 3D viewer. The time taken by each user to complete each task was recorded. After the test, the participants were interviewed to obtain the user’s test experience and suggestions.

#### 5.2.2. Results of Field Test

After completing the field test, a statistical analysis was performed based on the time users spent on each task, as shown in Table 6, Table 7 and Table 8. The four participants spent an average of approximately twenty-five seconds for Method 1, approximately thirteen seconds for Method 2, and an average of fifty seconds for Method 3. In the process of Method 1, both users C and D encountered an abnormal problem in the online facility management platform, resulting in the inability to complete Task 2 and 4 in Scenario 2. While User B was the most senior among the four participants, User B was familiar with using LINE communication software and had never used a facility management platform, which resulted in longer task execution times than the other three participants. User C had rich experience in operating the facility management platform and was familiar with using Line communication software, so they were the most efficient in executing tasks.

**Figure 14:**
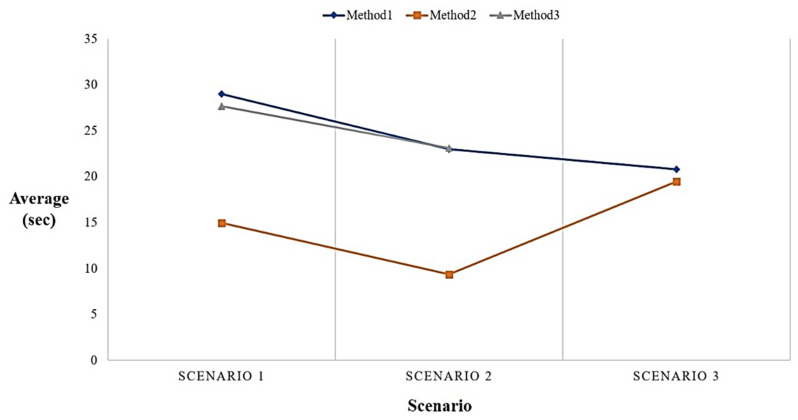
Statistical results of the three methods.

As shown in Figure 14, according to the statistical results, when the participant uses the clickable menu in the chatbot to retrieve information the efficiency is the highest and the speed is approximately twice as fast as the other two methods. Furthermore, a user had same the performance when entering natural language in the chatbot and using the facility management platform to retrieve information for Scenario 1 and 2. The reason for this result is that each user inputs text at different speeds, and without any search keywords, the user has difficulty in the search task. In addition, the results also show that the efficiency of using Method 1 and 2 in Scenario 3 is similar. This is because the chatbot and the facility management platform use the same model browser. Therefore, when the model browser is opened, the time spent in it does not differ much. By observing the user’s test process and analyzing the feedback given by the user, the following three conclusions of the field test can be summarized.

1. All participants agreed that it is more convenient and efficient to use the chatbot to retrieve facility information than to use a facility management platform and that the chatbot user interface is simpler and easier to learn.
2. It is necessary to add some tools in the 3D viewer to assist users in operating the 3D model, such as adding a model filter to simplify the number of models displayed. Otherwise, without the help of tools, users will be limited by the size of the mobile phone screen and have difficulty in operation.
3. Providing users with guidance or operation teaching can make using the dialogue system more smoothly. Especially under the lack of keyword guidance, users will be confused when they are asked to use natural language to search for information.

After completing the feasibility test and the field test, some feedback from these tests was obtained. The results obtained from the feasibility test allow the identification of weaknesses and areas for improvement in the system’s usability and test tasks. The field test results demonstrate putting the system into practice. Furthermore, it allows us to rethink how to design a more humanized design by observing the behavior of users operating the chatbot in the field test. The problems encountered by users, the questions they raised, and the subsequent solutions and improvement ideas will certainly help in delivering a better tool in the future.

## 6. Discussion

In this study, a conversation-based information delivery system for facility management was developed to solve the problem of low retrieval efficiency of traditional facility management platforms. According to the results of the feasibility test and field test, the developed chatbot was proven to assist facility engineers in retrieving equipment information and provides several contributions. However, by observing the user’s operation behavior and feedback, several limitations and challenges of chatbots were also found. The following sections will be divided into contributions and limitations of the proposed design for further detail.

### 6.1. Contributions

Based on the pre-test and field test results and user feedback, four contributions of the proposed conversation-based building information delivery system for facility management can be summarized:

#### 6.1.1. Simple Interface and Easy-to-Use Conversation-Based Delivery System

A chatbot was chosen for this delivery system as a chatbot can implement a simple interface, which makes the system easy to operate and reduces the operator error rate. By observing the user’s operation behavior in the feasibility test and field test, it was found that although the use of natural language to retrieve information in the chatbot is not efficient, young and old users can quickly get started conversing with the chatbot and can retrieve BIM information through a clickable menu.

#### 6.1.2. Personal Assistant for Providing Retrieval of BIM Information

By interviewing the needs of users to define the scope of tasks performed by the chatbots, a new BIM information retrieval system was built. In addition, the chatbot was built into the communication software of a mobile phone, so it is more portable than the traditional online platform, which will increase the interaction between the user and the dialogue system. Building on a chatbot also allows the user to obtain model information directly by using mobile devices, which replaces low-portability computer devices. Therefore, chatbots are more like a personal assistant than a facility management platform in helping users to get BIM information efficiently.

#### 6.1.3. Search Engine Based on Ontological Techniques

With a search engine developed by ontological techniques, structured BIM data is provided to the chatbot as a response database. This method helps users obtain information more accurately. When the user wants to query the equipment details for a certain floor, it is not necessary to filter through the layers, as the keywords containing the name of the floor and the equipment in a sentence can be recognized as the user’s true intention.

#### 6.1.4. Customized Features and Suitability for any BIM Model

The proposed delivery system based on dialogue can customize functions according to the operational habits of any user. Any BIM model is suitable for automatic data extraction and structuring in the knowledge base developed by this system. In addition to model information, the chatbot can also connect to any sensor API to provide users with real-time retrieval of information from on-site sensors. The retrieval of BIM data through the chatbot improves user efficiency in retrieving data, and this method can be used at any time.

### 6.2. Limitations

#### 6.2.1. Lack of Machine Learning Algorithms

The chatbot developed in this study is a retrieval-based model. The TF-IDF statistical and cosine similarity methods were used for keyword matching. This method lacks an algorithm based on machine learning and does not generate any new text. The algorithms select a response from a fixed set. Therefore, when there is no predefined response logic, the chatbot response logic will be wrong. In future work, machine learning algorithms for natural language processing (NLP) can be imported into the delivery system, such as using supervised machine learning models to automatically classify FM-related issues [48] to achieve a more mature response logic.

#### 6.2.2. High Labor Demand to Maintain the Corpus

In the developed chatbot, there is a corpus based on the results of user interviews. All words in this corpus need to be entered manually and thus it will require significant labor to add to and maintain the corpus when expanding the corpus in the future. In future work, the conversation records stored in the chatbot will be used to analyze users’ new, high frequency keywords to thus develop a module that can extract keywords into the corpus automatically.

#### 6.2.3. Incomplete Collection of Equipment Dataset

The field of facility management covers a wide range, such as electromechanical systems, firefighting systems, water supply and drainage systems, etc., so it is difficult to define a general dataset. This research only sets users in a part of facility engineers and defines the dataset through interviews. In future work, the research team will adopt relevant regulations or continuous interviews to expand the equipment dataset to serve more users.

### 6.3. Potential Applications of the System

The system architecture developed in this research can be applied not only to facility management, but also to other tasks and teams in the building. For example, the maintenance and operation of a building may require several security personnel to monitor the flow of people and notify of events, or the community committee needs to push an announcement to all residents, which can develop into a new dialogue through the architecture of this system. Regarding system construction, the new system only needs to define new datasets, dialogue logic, and interface design according to different users and import new BIM models into the ontological framework. The platform for implementing the dialogue system is not limited to fixed communication software as the chatbot can be transplanted according to the team’s communication software usage habits.

## 7. Conclusions

The lack of an effective information delivery method makes it difficult for FM-related personnel to retrieve required information and thus reduces the efficiency of the overall maintenance process. However, traditional FM platforms usually have drawbacks, such as complex user-interface and long data loading time, which lead to low information retrieval efficiency. In order to deliver the facility information in a more intuitive and efficient way, this research developed a novel information delivery method by utilizing natural language processing and ontology technologies. For implementation, a chatbot was prototyped based on a real administration building in Taiwan. The effectiveness and efficiency of the chatbot prototype was validated through both a feasibility test and a field test. The feasibility test results show that the chatbot can help the user complete different information retrieving tasks. The results of the field test show that using the rule-based approach with clickable chatbot interfaces can reduce the information retrieving time by 45.7% compared to the use of a traditional platform-based system. Results of this research validated the potential of using conversation-based systems to serve as a communication bridge between users and FM information. However, implementing a new method into a long-lasting manual or platform-based process is not a simple task. Studies focusing on how to help users apply new technology to their existing process should be conducted. Therefore, future works of this research will focus on new workflow definition, usability evaluation, and system improvement.

## Figures and Tables

**Table 1:** Previous studies that developed chatbots for information delivery.

Authors	Chatbot Name	Application Field	Purposes and Focuses
Oh et al. [33]	N/A	Mental health	Provide psychiatric counseling service in mental healthcare
Al-Zubaide and Issa [34]	OntBot	Not specified	Allow users to use chatbot with natural languages in different application domains
Vegesna et al. [43]	N/A	E-commerce	Provide users details of products on E-commerce website
Tsai et al. [44]	Ask Diana	Disaster Prevention	Serve as a management system that acquires, processes, and delivers disaster-related information
Cho and Lee [46]	N/A	Construction Management	Collect and process required information from users and generate daily reports automatically
Chan and Tsai [47]	N/A	Disaster Prevention	Serve as a decision support application in disaster management
Tsai et al. [11]	N/A	Building Inspection	Support assessors in performing school building safety inspections

**Table 2:** Equipment dataset for facility manager.

Building Name	Floor List	Room List	Equipment List	Attribute List
Name of the target building	B1F, 1F, 2F, …	Room 1, 2, 3, …	1. Exhaust fan 2. Cooling water tower 3. Air conditioner 4. Pump 5. Flow meter 6. Ice water host 7. Flowmeter sensor	1. Full code 2. Location information 3. Inspection record 4. Error codes 5. Detailed equipment information

**Table 3:** Designed tasks for the feasibility test.

	Method 1: Use Clickable Menu in the Chatbot to Execute Task	Method 2: Use Natural Language in the Chatbot to Execute Task
**Scenario 1: Use the chatbot to retrieve facility information**	The user is required to use the chatbot to retrieve facility information, including a list of equipment on specific floors, inspection records, the full code, and description of specific equipment.	
**Scenario 2: Use the chatbot to retrieve on-site sensor information**	The user is required to use the chatbot to retrieve the on-site sensor information, including the value and percentage of the thermal cumulative flow of the flow meter sensor on a specific floor.	
**Scenario 3: Use the chatbot to operate 3D viewer**	The user is required to use the chatbot to operate the 3D viewer and find a specific piece of equipment in the viewer.	N/A (3D viewer only supports clickable operations)

**Table 4:** Results of the feasibility test.

	Operation Time (s)							
	Method 1				Method 2			
	Participant A	Participant B	Participant C	Average Time	Participant A	Participant B	Participant C	Average Time
**Scenario 1**	142	72	104	106.00	143	106	110	119.67
**Scenario 2**	52	39	50	47.00	117	76	80	91.00
**Scenario 3**	12	20	15	15.67	N/A	N/A	N/A	N/A
**Total**	206	131	169	168.67	260	182	190	210.67
**Average**	68.67	43.67	56.33	56.22	130.00	91.00	95.00	105.33

**Table 5:** Designed tasks for the field test.

	Method 1: Use Existing FM Platform to Execute the Task	Method 2: Use Clickable Menus of the Chatbot to Execute the Task	Method 3: Use Natural Language with the Chatbot to Execute the Task
**Scenario 1:** **Retrieve facility information**	The user is required to use the chatbot to retrieve the following four facility information: (Task1): All equipment on the second floor (Task2): Inspection record of the flow meter on the fourth floor (Task3): All equipment on the fifth floor (Task4): The description of specific equipment on the sixth floor.		
**Scenario 2:** **Retrieve on-site sensor information**	The user is required to use the chatbot to retrieve the following four on-site sensors information: (Task1): The value of the thermal cumulative flow on the sixth floor. (Task2): The percentage of the thermal cumulative flow on the third floor. (Task3): The value of the thermal cumulative flow on the fourth floor. (Task4): The percentage of the thermal cumulative flow on the first floor.		
**Scenario 3:** **Operate 3D viewer**	The user is required to use the chatbot to operate the following 3D viewer tasks: (Task1): Successfully opened the 3D viewer. (Task2): Operate the three-dimensional viewer to find cooling tower model on the top floor in the viewer.		N/A (3D viewer only supports clickable operations)

**Table 6:** Field test results for Method 1.

		Operation Time (s)					
		A	B	C	D	Total	Average
**Scenario 1**	Task 1	16	38	18	13	465	29.06
	Task 2	46	47	18	21		
	Task 3	25	60	15	23		
	Task 4	25	52	22	26		
**Scenario 2**	Task 1	26	48	13	42	276	23.00
	Task 2	17	19	Failed	Failed		
	Task 3	17	42	12	14		
	Task 4	11	15	Failed	Failed		
**Scenario 3**	Task 1	12	13	6	24	167	20.87
	Task 2	25	56	12	19		
**Total**		220	390	116	182	908	N/A
**Average**		22.00	39.00	14.50	22.75	25.22	N/A

**Table 7:** Field test results for Method 2.

		Operation Time (s)					
		A	B	C	D	Total	Average
**Scenario 1**	Task 1	9	25	9	10	241	15.00
	Task 2	13	27	12	13		
	Task 3	10	30	10	14		
	Task 4	12	20	14	13		
**Scenario 2**	Task 1	6	19	10	11	150	9.37
	Task 2	3	14	9	14		
	Task 3	7	12	7	9		
	Task 4	4	11	4	10		
**Scenario 3**	Task 1	12	9	6	6	156	19.50
	Task 2	25	34	30	34		
**Total**		101	201	111	134	547	N/A
**Average**		10.10	20.10	11.10	13.40	13.675	N/A

**Table 8:** Field test results for Method 3.

		Operation Time (s)					
		A	B	C	D	Total	Average
**Scenario 1**	Task 1	9	19	16	10	443	27.68
	Task 2	18	30	26	37		
	Task 3	23	39	98	57		
	Task 4	14	20	15	12		
**Scenario 2**	Task 1	13	38	22	20	370	23.125
	Task 2	10	52	23	23		
	Task 3	7	38	15	21		
	Task 4	13	40	17	18		
**Total**		107	276	232	198	813	N/A
**Average**		10.70	27.60	23.20	19.80	50.81	N/A

## Data Availability

Not applicable.

## References

[1] Oti A.H., Kurul E., Cheung F., Tah J.H.M. (2016). A framework for the utilization of Building Management System data in building information models for building design and operation. Autom. Constr..

[2] Lee W.L., Tsai M.H., Yang C.H., Juang J.R., Su J.Y. (2016). V3DM+: BIM interactive collaboration system for facility management. Vis. Eng..

[3] Chen L., Shi P., Tang Q., Liu W., Wu Q. (2020). Development and application of a specification-compliant highway tunnel facility management system based on BIM. Tunn. Undergr. Space Technol..

[4] Lin Y.C., Su Y.C., Chen Y.P. (2014). Developing mobile BIM/2D barcode-based automated facility management system. Sci. World J..

[5] Chan D.W.M. (2019). Sustainable building maintenance for safer and healthier cities: Effective strategies for implementing the Mandatory Building Inspection Scheme (MBIS) in Hong Kong. J. Build. Eng..

[6] Kwon N., Song K., Ahn Y., Park M., Jang Y. (2020). Maintenance cost prediction for aging residential buildings based on case-based reasoning and genetic algorithm. J. Build. Eng..

[7] Chai C., de Brito J., Gaspar P.L., Silva A. (2014). Predicting the service life of exterior wall painting: Techno-economic analysis of alternative maintenance strategies. J. Constr. Eng. Manag..

[8] Prieto A.J., Silva A., de Brito J., Macías-Bernal J.M., Alejandre F.J. (2017). Multiple linear regression and fuzzy logic models applied to the functional service life prediction of cultural heritage. J. Cult. Herit..

[9] Tavares J., Silva A., de Brito J. (2020). Computational models applied to the service life prediction of External Thermal Insulation Composite Systems (ETICS). J. Build. Eng..

[10] Tsai M.H., Yang C.H., Chen J.Y., Kang S.C. (2021). Four-stage framework for implementing a chatbot system in disaster emergency operation data management: A flood disaster management case study. KSCE J. Civ. Eng..

[11] Tsai M.H., Chan H.Y., Liu L.Y. (2020). Conversation-based school building inspection support system. Appl. Sci..

[12] Wu E.H.K., Lin C.H., Ou Y.Y., Liu C.Z., Wang W.K., Chao C.Y. (2020). Advantages and constraints of a hybrid model K-12 E-Learning assistant chatbot. IEEE Access..

[13] Tsai M.H., Mom M., Hsieh S.H. (2014). Developing critical success factors for the assessment of BIM technology adoption: Part I. Methodology and survey. J. Chin. Inst. Eng..

[14] Akcamete A., Liu X., Akinci B., Garrett J.H. Integrating and visualizing maintenance and repair work orders in BIM: Lessons learned from a prototype. Proceedings of the 11th International Conference On Construction Applications of Virtual Reality (CONVR, 2011).

[15] Becerik-Gerber B., Jazizadeh F., Li N., Calis G. (2012). Application areas and data requirements for BIM-enabled facilities management. J. Constr. Eng. Manag..

[16] Kelly G., Serginson M., Lockley S., Dawood N., Kassem M. BIM for facility management: A review and a case study investigating the value and challenges. Proceedings of the 13th International Conference on Construction Applications of Virtual Reality (CONVR, 2013).

[17] Kassem M., Kelly G., Dawood N., Serginson M., Lockley S. (2015). BIM in facilities management applications: A case study of a large university complex. Built Environ. Proj. Asset Manag..

[18] Lee S.K., An H.K., Yu J.H. An Extension of the technology acceptance model for BIM-based FM. Proceedings of the Construction Research Congress 2012: Construction Challenges in a Flat World.

[19] Akcamete A., Akinci B., Garrett J.H. Potential utilization of building information models for planning maintenance activities. Proceedings of the International Conference on Computing in Civil and Building Engineering (ICCCBE, 2010).

[20] Eastman C.M., Teicholz P., Sacks R., Liston K. (2011). BIM Handbook: A guide to building information modeling for owners. Managers, Architects, Engineers, Contractors, and Fabricators.

[21] Edirisinghe R., London K.A., Kalutara P., Aranda-Mena G. (2017). Building information modelling for facility management: Are we there yet?. Eng. Constr. Archit. Manag..

[22] Yalcinkaya M., Singh V. (2014). Building information modeling (BIM) for facilities management-literature review and future needs. IFIP Adv. Inf. Commun. Technol..

[23] Nepal M.P., Staub-French S., Pottinger R., Zhang J. (2013). Ontology-based feature modeling for construction information extraction from a building information model. J. Comput. Civ. Eng..

[24] Lin J.R., Hu Z.Z., Zhang J.P., Yu F.Q. (2016). A Natural-Language-Based Approach to Intelligent Data Retrieval and Representation for Cloud BIM. Comput. Civ. Infrastruct. Eng..

[25] Zhang L., Issa R.R.A. (2013). Ontology-based partial building information model extraction. J. Comput. Civ. Eng..

[26] Pärn E.A., Edwards D.J. (2017). Conceptualising the FinDD API plug-in: A study of BIM-FM integration. Autom. Constr..

[27] Ergen E., Akinci B., Sacks R. (2007). Life-cycle data management of engineered-to-order components using radio frequency identification. Adv. Eng. Inform..

[28] Chen G., Luo Y. A BIM and ontology-based intelligent application framework. Proceedings of the 2016 IEEE Advanced Information Management, Communicates, Electronic and Automation Control Conference (IMCEC, 2016).

[29] Liu H., Lu M., Al-Hussein M. (2016). Ontology-based semantic approach for construction-oriented quantity take-off from BIM models in the light-frame building industry. Adv. Eng. Inform..

[30] Kiviniemi A., Codinhoto R. Challenges in the implementation of BIM for FM—Case Manchester town hall complex. Proceedings of the 2014 International Conference on Computing in Civil and Building Engineering (ICCCBE, 2014).

[31] Zhang C., Beetz J., DeVries B. (2018). BimSPARQL: Domain-specific functional SPARQL extensions for querying RDF building data. Semant. Web..

[32] Katranuschkov P., Gehre A., Scherer R.J. (2003). An ontology framework to access IFC model data. Electron. J. Inf. Technol. Constr..

[33] Niknam M., Karshenas S. (2017). A shared ontology approach to semantic representation of BIM data. Autom. Constr..

[34] Lee S.K., Kim K.R., Yu J.H. (2014). BIM and ontology-based approach for building cost estimation. Autom. Constr..

[35] Chen W., Chen K., Cheng J.C. (2006). Towards an Ontology-based Approach for Information Interoperability Between BIM and Facility Management. Proceedings of the Workshop of the European Group for Intelligent Computing in Engineering.

[36] Wang H.H., Boukamp F., Elghamrawy T. (2011). Ontology-based approach to context representation and reasoning for managing context-sensitive construction information. J. Comput. Civ. Eng..

[37] Karshenas S., Niknam M. Ontology-based building information modeling. Proceedings of the 2013 ASCE International Workshop on Computing in Civil Engineering (IWCC, 2013).

[38] Zhang L., Issa R.R.A. Development of IFC-based construction industry ontology for information retrieval from IFC models. Proceedings of the 2011 Eg-Ice Workshop.

[39] Jiang S., Zhang J. Development of an ontology-based semantic retrieval method for construction project risk management. Proceedings of the 2013 International Conference on Construction and Real Estate Management (ICCREM, 2013).

[40] Nuruzzaman M., Hussain O.K. (2020). IntelliBot: A Dialogue-based chatbot for the insurance industry. Knowl. Based Syst..

[41] Oh K.J., Lee D., Ko B., Choi H.J. A chatbot for psychiatric counseling in mental healthcare service based on emotional dialogue analysis and sentence generation. Proceedings of the 18th IEEE International Conference on Mobile Data Management.

[42] Al-Zubaide H., Issa A.A. OntBot: Ontology based ChatBot. Proceedings of the 4th International Symposium on Innovation in Information and Communication Technology (ISIICT 2011).

[43] Vegesna A., Jain P., Porwal P. (2018). Ontology based Chatbot (For E-commerce Website). Int. J. Comput. Appl..

[44] Tsai M.H., Chen J.Y., Kang S.C. (2019). Ask Diana: A keyword-based chatbot system for water-related disaster management. Water.

[45] Tsai M.H., Chan H.Y., Chan Y.L., Shen H.K., Lin P.Y., Hsu J.W. (2021). A Chatbot System to Support Mine Safety Procedures during Natural Disasters. Sustainability.

[46] Cho J., Lee G. A chatbot system for construction daily report information management. Proceedings of the 36th International Symposium on Automation and Robotics in Construction (ISARC 2019).

[47] Chan H.Y., Tsai M.H. (2019). Question-answering dialogue system for emergency operations. Int. J. Disaster Risk Reduct..

[48] McArthur J.J., Shahbazi N., Fok R., Raghubar C., Bortoluzzi B., An A. (2018). Machine learning and BIM visualization for maintenance issue classification and enhanced data collection. Adv. Eng. Inform..

[49] Manning D., Raghavan P., Schtze H. (2008). Introduction to Information Retrieval.

[50] Tata S., Patel J.M. (2007). Estimating the selectivity of tf-idf based cosine similarity predicates. SIGMOD Rec..

[51] Autodesk Inc. Revit. https://www.autodesk.com/products/revit/overview.

[52] The Board of Trustees of the Leland Stanford Junior University Protégé. https://protege.stanford.edu/.

[53] Alatrish S. (2021). Comparison of Ontology Editors. E-RAF J. Comput..

[54] Microsoft, C# Documentation. https://docs.microsoft.com/en-gb/dotnet/csharp/.

[55] Autodesk Inc. Online Documentation for the Revit API. https://www.revitapidocs.com/.

[56] Python Software Foundation Project Description. https://www.python.org/psf/.

[57] Autodesk Inc. Dynamo. https://dynamobim.org/.

[58] The Knowledge Representation and Reasoning Group. http://www.hermit-reasoner.com/.

[59] Taiwan Network Information Center 2018 Taiwan Internet Report. https://www.twnic.net.tw/doc/twrp/201812e.pdf.

